# Progress in the mechanism of functional dyspepsia: roles of mitochondrial autophagy in duodenal abnormalities

**DOI:** 10.3389/fmed.2024.1491009

**Published:** 2024-11-25

**Authors:** Kexin Zhong, Xiaojuan Du, Yuanyuan Niu, Zhengju Li, Yongbiao Tao, Yuqian Wu, Ruiting Zhang, Linjing Guo, Yurong Bi, Lijuan Tang, Tianyu Dou, Longde Wang

**Affiliations:** ^1^Clinical College of Traditional Chinese Medicine, Gansu University of Traditional Chinese Medicine, Lanzhou, China; ^2^Key Laboratory of Cell Activities and Stress Adaptations, Ministry of Education, School of Life Sciences, Lanzhou University, Lanzhou, China; ^3^College of Pharmacy, Gansu University of Traditional Chinese Medicine, Lanzhou, China; ^4^College of Life Sciences, China Jiliang University, Hangzhou, China; ^5^Department of Gastroenterology, Affiliated Hospital of Gansu University of Chinese Medicine, Lanzhou, China

**Keywords:** duodenal abnormalities, functional dyspepsia, mitochondrial autophagy, pathogenesis, gastrointestinal dysfunction

## Abstract

Mitochondria are the main source of energy for cellular activity. Their functional damage or deficiency leads to cellular deterioration, which in turn triggers autophagic reactions. Taking mitochondrial autophagy as a starting point, the present review explored the mechanisms of duodenal abnormalities in detail, including mucosal barrier damage, release of inflammatory factors, and disruption of intracellular signal transduction. We summarized the key roles of mitochondrial autophagy in the abnormal development of the duodenum and examined the in-depth physiological and pathological mechanisms involved, providing a comprehensive theoretical basis for understanding the pathogenesis of functional dyspepsia. At present, it has been confirmed that an increase in the eosinophil count and mast cell degranulation in the duodenum can trigger visceral hypersensitive reactions and cause gastrointestinal motility disorders. In the future, it is necessary to continue exploring the molecular mechanisms and signaling pathways of mitochondrial autophagy in duodenal abnormalities. A deeper understanding of mitochondrial autophagy provides important references for developing treatment strategies for functional dyspepsia, thereby improving clinical efficacy and patient quality of life.

## 1 Introduction

Functional dyspepsia (FD) is a chronic gastrointestinal disease that occurs in the gastroduodenal region, and it is characterized by epigastric pain, burning sensation, postprandial satiety, or early satiety. According to the Roman IV standard, patients can be classified into two subgroups based on their specific symptoms: postprandial distress syndrome (PDS) and epigastric pain syndrome (EPS) (1), with PDS being more common than EPS. The global prevalence of FD ranges from 10 to 30% (2), and only 40% of patients with FD seek medical assistance when symptoms worsen or occur frequently (3). Although FD is a non-fatal disease, due to its prolonged course and overlap with irritable bowel syndrome (IBS) and gastroesophageal reflux disease (GERD), long-term FD not only increases the incidence of depression, anxiety, and sleep disorders but may also lead to gastrointestinal bleeding and iron deficiency anemia (2), which reduces the patient’s quality of life and increases medical costs, causing serious economic losses and social burden (4).

The pathogenesis of FD is not yet fully understood. Current known pathological factors involved in FD pathogenesis include inflammation, gastrointestinal motility disorders, visceral hypersensitivity reactions, gut-brain axis disorders, and psychological disorders.

It is currently believed that these mechanisms are not completely independent but interact and influence each other. This mutual interaction ultimately leads to gut-brain axis abnormalities, which are important mechanisms involved in the development and progression of FD. Research has found that patients with FD exhibit changes in the activity of certain brain regions, including the frontal cortex, the somatosensory cortex, the insula, and the anterior cingulate cortex. These activity changes are associated with dyspeptic symptoms, anxiety, and depression, indicating that patients with FD are prone to dysfunction in brain regions involved in sensory and pain modulation, as well as emotion and homeostasis regulation (5).

With in-depth research on FD, an increasing amount of evidence suggests that duodenal abnormalities are among the important causes of the disease (6–8).

Mitochondrial autophagy can be stimulated by various cellular organelles. Stress-induced changes in the structural proteins of the Golgi apparatus, including GM130, result in fragmentation. This fragmentation is commonly linked to an elevation in intracellular calcium ion levels, which subsequently impairs mitochondrial function. Increased calcium ions can alter mitochondrial membrane permeability, leading to a decline in mitochondrial membrane potential (ΔΨm) and an upregulation of reactive oxygen species (ROS) production, thereby initiating mitochondrial autophagy (9). The interplay between the Golgi, endoplasmic reticulum, and mitochondria is pivotal in this process. The Golgi apparatus can transfer specific signaling proteins, such as BNIP3L/NIX, to mitochondria, aiding in the identification of damaged mitochondria and their entry into the autophagic pathway (10). Lysosomes are tasked with degrading mitochondrial contents, representing the terminal phase of mitochondrial autophagy. Upon engulfment by autophagosomes, lysosomes fuse with these structures to decompose and degrade the compromised mitochondrial components (9). Autophagy receptors, including p62 and NBR1, interact with specific markers to ensure the recognition and delivery of damaged mitochondria to lysosomes for degradation. ROS production directly influences lysosomal function and further stimulates the autophagic pathway. In cellular injury responses, ROS function as signals, enhancing lysosomal autophagy activity by activating specific signaling pathways, such as the NF-κB pathway (10).

Research indicates that gastrointestinal interstitial cells of Cajal (ICC) serve as pacemaker cells for gastrointestinal motility, influencing mitochondrial function through the regulation of Ca^2+^ fluctuations and thereby modulating the contraction and relaxation of intestinal smooth muscles (11). An imbalance in Ca^2+^ metabolism can trigger endoplasmic reticulum stress (ERs), which, by activating abnormal mitophagy, leads to mitochondrial damage. Impaired mitophagy results in the failure to clear damaged mitochondria, further exacerbating ICC apoptosis, and disrupting gastrointestinal electrical rhythms and smooth muscle contractility (12). Thus, mitophagy and apoptosis play crucial roles in regulating intestinal homeostasis and reducing gastrointestinal damage (13).

When eosinophils and mast cells in the duodenum are activated, mitophagy, a specific autophagic mechanism, helps to remove excessive or damaged mitochondria to maintain mitochondrial balance in terms of quantity and function. This process plays a key role in regulating the number of mitochondria within the cell and ensuring their proper function, contributing to the stability of the gastrointestinal environment (14). Although the role of mitophagy in the gastrointestinal tract has not been extensively studied, existing research has shown that mitochondrial redox imbalances can generate excessive free radicals, affecting duodenal mucosal permeability and physiological function (15). Therefore, further investigation into the impact of mitophagy on duodenal dysfunction could help elucidate the pathogenesis of FD and provide more effective strategies for its prevention and treatment.

## 2 Mitophagy

Mitochondria are present in most eukaryotic cells and are composed of an outer membrane (OMM), an intermembrane space, an inner membrane (IMM), and a matrix (16). As an “energy reservoir,” mitochondria provide energy for the cellular biochemical reactions required for life and serve as a hub for cellular signal transduction (17). In addition to supplying energy for the biochemical reactions necessary for life, mitochondria also serve as a central hub for cellular signaling, playing an essential role in processes such as cell growth, differentiation, and apoptosis (18). In 1966, De Duve and Wattiaux (19) referred to the transmission of cellular components through autophagosomes as “autophagy.” Based on the mechanism in mammalian cells, autophagy can be classified into three different types: microautophagy, chaperone-mediated autophagy (CMA), and macroautophagy (20). Microautophagy involves the direct engulfment and degradation of cellular contents in lysosomes or late endosomes without changes to membrane structure (21). CMA has high selectivity, involving the degradation of only damaged and mutated proteins without vesicle involvement (22). Macroautophagy involves the transportation of cytoplasmic components into lysosomes through the double-layered membrane-binding vesicles in the middle. When autophagosomes bind to lysosomes, they generate autophagic lysosomes (23). Another classification of autophagy is based on differences in substrate degradation, and it classifies autophagy as non-selective autophagy and selective autophagy. In non-selective autophagy, proteins or organelles are randomly transferred to lysosomes for decomposition. Selective autophagy is the process by which cells maintain their structural stability by actively ingesting certain specific proteins and organelles. The substrates that autophagy degrades have specificity, and mitochondrial autophagy is a type of selective autophagy. In 2005, selective mitochondrial autophagy was first termed “mitochondrial autophagy,” clearly indicating that mitochondrial damage is a hallmark of the initiation of mitophagy (24).

## 3 Pathways of mitochondrial autophagy

When cells are stimulated, mitochondria undergo depolarization, and the “eat me” molecular signals on their surface are recognized by autophagy mechanisms, ensuring that only specific mitochondria are targeted for degradation (25–27). At present, the mechanisms of mitochondrial autophagy mainly include two categories: ubiquitin-mediated mitophagy (Ub) and non-ubiquitin mitophagy.

### 3.1 The ubiquitin-dependent pathway

**Figure 1:**
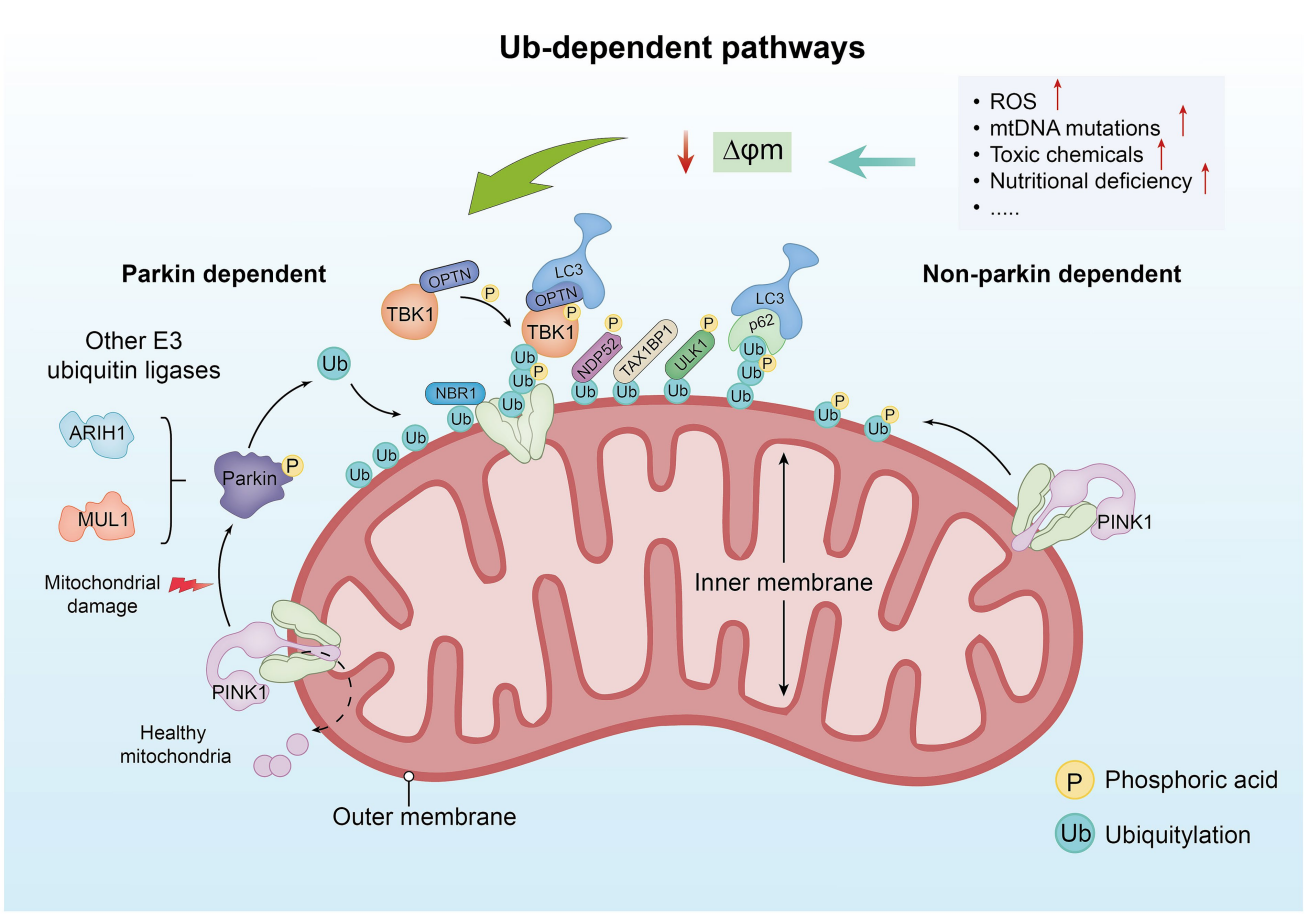
The ubiquitin-dependent pathway. In healthy mitochondria, PINK1 is typically transported from the outer membrane to the inner membrane and rapidly degraded. However, when factors such as increased ROS levels, mitochondrial DNA mutations, toxic substances, and nutrient deficiencies lead to damage of the ΔΨm, PINK1 accumulates on the outer membrane of damaged mitochondria and forms dimers, which are activated through autophosphorylation. Under conditions of mitochondrial damage, PINK1 fails to be transported to the inner membrane and begins to accumulate on the outer membrane. Upon accumulation, PINK1 phosphorylates Ub and Parkin, activating the ubiquitination process. Parkin, an E3 ubiquitin ligase, recognizes damaged mitochondria and facilitates the ubiquitination of proteins on the mitochondrial surface. The ubiquitinated mitochondria are further modified by other E3 ubiquitin ligases (such as ARIH1 and MUL1), forming additional ubiquitin chains that aid in the recruitment of downstream autophagy-related proteins. Proteins such as p62, NBR1, TAX1BP1, OPTN, and NDP52 are recruited to ubiquitinated mitochondria, promoting their recognition and engulfment by autophagosomes, thereby completing the process of mitophagy. Non-Parkin-dependent mitophagy primarily relies on PINK1. In this pathway, PINK1 accumulates on the mitochondrial membrane and induces autophagy. The phosphorylation activity of PINK1 is related to the ubiquitination pathway, assisting in the recognition of damaged mitochondria and initiating their clearance.

In Ub-mediated mitochondrial autophagy, the PTEN-induced kinase 1/Parkin RBR E3 ubiquitin protein ligase (PINK1/Parkin) signaling pathway is one of the current research hotspots (28). When the mitochondrial function is normal, PINK1 is continuously transported to mitochondria, where it is rapidly degraded to maintain its low level within the cell. When the ΔΨm is impaired, PINK1 accumulates on the outer membrane of damaged mitochondria, forming dimers that are activated through self-phosphorylation. In this state, the intramolecular structure of Parkin changes, releasing ubiquitin-like substances (Ubl), which are easily phosphorylated by PINK1 at the Ser65 site (29). When Parkin is activated, it catalyzes the generation of ubiquitin chains on the outer membrane proteins of damaged mitochondria. These ubiquitin chains serve as binding sites for autophagy adapter proteins, including sequestosome 1 (SQSTM1/p62), next to BRCA1 gene 1 (NBR1), Tax1 binding protein 1 (TAX1BP1), optineurin (OPTN), and nuclear dot protein 52 (NDP52), further inducing the formation of autophagosomes and promoting the progression of mitochondrial autophagy (30, 31) (Figure 1). Among these proteins, OPTN, TAX1BP1, and NDP52 are the main driving factors (28). OPTN is highly expressed in brain tissues and is an important “carrier adapter” that often plays a role in neurodegenerative diseases (32). TAX1BP1 was initially discovered through yeast two-hybrid screening (33). Its structure is complex, including a SKIP carboxyl homology (SKICH), three spiral coiled regions, and a zinc finger domain (34). NDP52 triggers PINK1/Parkin mitochondrial autophagy by recruiting ULK1/2 protein kinase complexes to guide damaged mitochondria (35). p62 is a scaffold protein composed of 440 amino acids that is involved in various functions, including autophagy, apoptosis, and inflammation. Studies have confirmed that the expression of p62 is increased in septic rats injected with dl-3-n-butylphthalide (NBP). NBP ameliorated intestinal microcirculation disorders and damage to small intestinal vascular endothelial cells in septic rats by activating the PI3K/Akt signaling pathway and regulating mitochondrial autophagy, thereby alleviating symptoms of sepsis. This experiment indirectly suggests that p62 may be associated with intestinal inflammation (36). Studies have also indicated that p62 acts as an effector in the induction of apoptosis during chemotherapy in colorectal cancer cells, with increased p62 levels potentially enhancing chemotherapy sensitivity (37). NBR1 has a similar domain as p62 and is more widely distributed than p62. Research has shown that the overexpression of NBR1 can promote the metastasis and growth of tumor cells (38). Furthermore, research has indicated that besides Parkin, other E3 ubiquitin ligases are also involved in the ubiquitination of mitochondrial surface proteins, such as mitochondrial anchored protein ligase 1 (MUL1) and ariadne-1 homolog (ARIH1) (39, 40).

An experimental study generated an MUL1A6 mutant by knocking out the P element in the MUL1 coding gene of *Drosophila* (41). They observed that the mitochondria of fruit flies carrying this mutant type became smaller and fragmented, suggesting that MUL1 may be associated with the process of mitochondrial lysis. A recent study confirms that MUL1 can compensate for mutations in PINK1 or Parkin by reducing mitochondrial fusion, thereby maintaining mitochondrial integrity (28). In 2023, a study found that acyl acids can cause an overexpression of MUL1 and, thus, lessen the decrease in mitochondrial fusion protein (Mfn2) and enhance the increase of mitochondrial related protein 1 (Drp1) levels, thereby inhibiting mitochondrial fragmentation (42). Another study demonstrated that non-Parkin-dependent mitophagy primarily relies on PINK1 (43). In this pathway, PINK1 first accumulates on the mitochondrial membrane and then induces autophagy. PINK1’s role extends beyond activating mitophagy because its phosphorylation activity is also closely linked to the ubiquitination pathway. This phosphorylation enables PINK1 to effectively recognize damaged mitochondria, thereby initiating their clearance. In this way, PINK1 ensures mitochondrial quality control within the cell, promoting cellular health and normal function by preventing dysfunction caused by the accumulation of damaged mitochondria.

### 3.2 The non-ubiquitin-dependent pathway

In the non-ubiquitin-dependent pathway, specific receptor proteins on the outer membrane of mitochondria directly bind to autophagy microtubule-associated protein 1 light chain 3 (LC3) without undergoing ubiquitination, thereby achieving mitochondrial autophagy.

**Figure 2:**
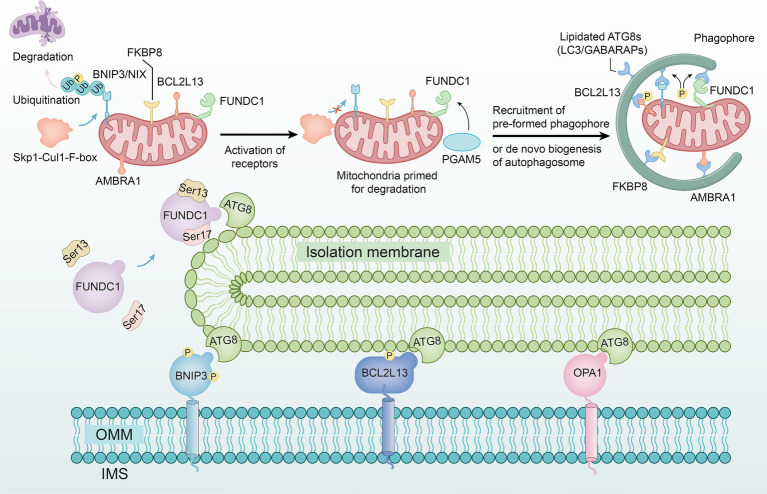
The non-ubiquitin-dependent pathway. The SCF complex formed by the F-box and FBXL4 genes maintains the stability of BNIP3 and NIX on the outer mitochondrial membrane, preventing their accumulation and homodimerization, thereby inhibiting their interaction with ATG8 and phosphorylation to regulate mitochondrial autophagy. FUNDC1 undergoes phosphorylation under hypoxic conditions and interacts with ATG8 proteins on damaged mitochondria, mediating mitochondrial autophagy. BCL2L13 regulates mitochondrial morphology, with its overexpression inducing fission and its depletion causing elongation, potentially inducing autophagy. Interaction between FKBP8 and OPA1 disrupts the stability of mitochondrial dynamics. AMBRA1 can directly trigger mitochondrial autophagy, not only by enhancing Parkin-independent autophagy through promoting PINK1 stability but also by inducing its localization to mitochondria via optogenetics.

Research has shown that Skp1-Cul1-F-box (SCF) mediated by F-box and leucine rich repeat protein 4 (FBXL4) is located on the outer mitochondrial membrane and can maintain B-cell leukemia/lymphoma-2 (BCL-2), BCL-2/adeno‐virus E1B 19 k D interacting protein 3 (BNIP3), and NIP3-like protein X (NIX) at lower levels (44). When NIX and BNIP3 continuously accumulate on the outer membrane, they undergo homologous dimerization and regulate mitochondrial autophagy through interaction with and phosphorylation of the autophagy related gene 8 (ATG8) (45) (Figure 2).

The FUN14 domain containing protein 1 (FUNDC1) is a novel mitochondrial membrane protein that mediates the autophagy process of mitochondria (46). Under hypoxic conditions, FUNDC1 activates the dephosphorylation of Ser13 mediated by phosphoglycerate mutase 5 (PGAM5) and the phosphorylation of Ser17 mediated by unc-51 like kinase 1 (ULK1), thereby interacting with the ATG8 family proteins on damaged mitochondria (47) (Figure 2).

BCL-2-like protein 13 (BCL2L13) is a member of the BCL-2 family. Studies have shown that overexpression of BCL2L13 leads to mitochondrial division, while loss of BCL2L13 leads to mitochondrial elongation, indicating that BCL2L13 may induce autophagy by affecting mitochondrial morphology (48) (Figure 2).

FK506 binding protein 8 (FKBP8) serves as an LC3-interacting protein. When it interacts with optic atrophy 1 (OPA1), mitochondrial dynamic homeostasis is disrupted by dynamin-related protein 1 (Drp1) (49) (Figure 2).

Autophagy/becklin-1 regulator 1 (AMBRA1) was initially recognized as a Parkin-interacting protein (50), is highly expressed in neural cells, and is capable of promoting Parkin-independent mitochondrial autophagy by enhancing the stability of PINK1. However, studies have demonstrated that using methods such as photogenetics to induce AMBRA1 localization on mitochondria, mitochondrial autophagy can be directly triggered (51) (Figure 2).

## 4 Pathological mechanism of duodenal abnormalities

At present, the exact cause of duodenal abnormalities in FD is still unknown. Relevant factors include external stimuli, gut microbiota, bile acid (BA) metabolism disorders, duodenal acid, and emotional disorders (52, 53).

Typically, small amounts of ${HCO}_{3}^{-}$ neutralize H^+^ ions that diffuse back through the mucus layer, thereby protecting the duodenal mucosa from excessive acid. When the duodenum of patients with FD is stimulated by external stimuli, the secretion of ${HCO}_{3}^{-}$ by epithelial cells decreases, resulting in intestinal acidification, disruption of acid–base balance, and subsequent damage to the duodenal mucosal barrier (54), which makes the duodenum more susceptible to further external insults, exacerbating visceral hypersensitivity symptoms.

The latest research shows that the gut microbiota activates Toll like receptor 2 (TLR2) signaling in the intestinal epithelium and inhibits neuropilin 1 (NRP1) and its regulated Hedgehog (Hh) signaling in the intestinal epithelium, thereby weakening the function of the intestinal epithelial barrier (55). In addition, during the process of infection by intestinal pathogens, the gut microbiota can form a bacterial membrane barrier and promote the proliferation and differentiation of intestinal epithelial cells, forming a protective barrier to maintain immune homeostasis (56). These mechanisms may play a key role in gastrointestinal inflammation and functional diseases induced by mucosal T cells (57). A study proposed that dysbiosis of gut microbiota might affect the mental health of patients with FD, induce mental illness, and accelerate the course of FD (58).

BAs are mainly responsible for regulating the digestion and uptake of substances such as cholesterol, triglycerides, and fat-soluble vitamins (59). In recent years, it has been recognized that BAs can also function as signaling molecules (60). Research has shown that BAs regulate epithelial cell proliferation, gene expression, and energy metabolism by activating multiple receptors, including the vitamin D receptor (VDR), pregnane X receptor (PXR), farnesoid X receptor (FXR), and takeda G protein-coupled receptor 5 (TGR5). These receptors are distributed in the intestine, liver, muscles, central nervous system, and peripheral nervous system, mediating signal cascades and activating inflammatory factors (61). Studies have demonstrated that the VDR regulates immunity, intestinal barrier integrity, and inflammation (62–64). Upon stimulation and binding by vitamin D (VD) or lithocholic acid (LCA), VDR influences the growth, differentiation, and physiological functions of intestinal cells by inhibiting NF-κB signaling, thereby exerting anti-inflammatory effects on colon cancer cells (65). This includes maintaining the integrity of the intestinal mucosal barrier structure, regulating the balance of the gut microbiota, and modulating the function of the intestinal immune system. Damage to the intestinal mucosal barrier or microbiota imbalance can lead to the production and release of inflammatory factors. Furthermore, VD and VDR are involved in regulating the intestinal immune system’s function. For example, VD3 acts on antigen-presenting cells, inhibiting their ability to present antigens and activate T-cell immune responses. This regulation affects the production and release of inflammatory factors, such as interleukins (ILs), tumor necrosis factors (TNFs), and interferons (IFNs), that play crucial roles in intestinal inflammation and immune responses. PXR, a member of the nuclear receptor superfamily NR1I2, primarily functions to detect the presence of toxic substances and subsequently upregulate the expression of proteins involved in detoxification and clearance. While PXR is critical in drug metabolism and detoxification, it does not directly mediate signaling cascades that activate inflammatory factors. FXR, also known as the BA receptor, is another member of the nuclear receptor superfamily, and it is predominantly expressed in the liver and small intestine. FXR regulates BA synthesis, homeostasis, and transport through binding to specific ligands, which are mainly BAs. Studies have shown that FXR deficiency affects the homeostatic regulation of BAs as well as lipid and glucose metabolism (66–69). In the gastrointestinal tract, FXR influences inflammation by regulating BA metabolism and inhibiting inflammatory signaling pathways. FXR has also been shown to reduce lipid accumulation, which can induce the formation of ROS and activate NF-κB-related pathways, exacerbating inflammation (70–72). TGR5 is widely expressed in various tissues, including those of the gastrointestinal system, and plays a significant role in regulating lipid and glucose metabolism. As a membrane receptor, TGR5 can bind ligands and internalize them into the cytoplasm, leading to changes in various intracellular pathways and exerting significant regulatory effects. In muscle and adipose tissue, TGR5 increases thermogenesis, influencing mitochondrial energy homeostasis (73, 74). Additionally, TGR5 promotes the release of glucagon-like peptide 1 (GLP-1) from enteroendocrine cells in the gut, which enhances insulin secretion (75, 76). Moreover, TGR5 reduces inflammation in Kupffer cells (KCs) and in the intestine, which is related to inflammatory bowel disease (IBD), by inhibiting NF-κB nuclear translocation (77–79).

Eosinophils (EOS) play a crucial role in the pathological mechanism of diseases of the duodenum. EOS are activated by eotaxin through the CC chemokine receptor type 3 (CCR3) and function in conjunction with other receptors. When integrin α4 or β7 on the EOS surface binds to its specific receptor, it leads to the release of recombinant mucosal addressin cell adhesion molecule 1 (Madcam1) by endothelial cells of the intestinal mucosal lamina propria venules into the blood, thereby inducing an inflammatory response in the duodenum. In addition to the increase in the number of EOS, multiple clinical studies have also shown the degranulation of eosinophils in pediatric and adult patients with FD (80, 81), making the pathological process of FD in the duodenum more complex.

In FD, eosinophilic esophagitis (EoE) provides certain reference values for the specific role of EOS in duodenal abnormalities. In EoE, the immune response triggered by food antigens is closely related to esophageal barrier dysfunction, which activates mast cells and B cells (82). Similarly, EOS in the duodenum may be activated by IL-5, and they may activate mast cells through the secretion of myelin basic protein (MBP) from specific granules. Due to the involvement of EOS in the process of epithelial cell damage and repair, EOS degranulation may also lead to changes in duodenal permeability (83), and IL-5 is considered the main helper Th2 cytokine that leads to EOS activation (84). In patients with FD, the increase in IL-5 and IL-13 levels also indicates that this process has Th2-type immune response characteristics (85), which is very similar to the increased expression of IL-5 in CD4^+^ T cells in patients with EoE (86). These research findings suggest that the Th2-type immune response induced by EOS may play an important role in the pathogenesis of FD.

## 5 The role of mitochondrial autophagy in duodenal abnormalities

Mitochondria-induced autophagy is a cellular self-protective mechanism aimed at removing damaged or excess mitochondria to maintain intracellular homeostasis. Through this process, cells can recycle useful components from mitochondria and prevent damaged mitochondria from releasing harmful substances that could further impair cellular structure and function. When mitochondria are damaged, they send signals to activate autophagy-related proteins within the cell. These proteins facilitate the formation of autophagosomes, which encapsulate and isolate the damaged mitochondria. The autophagosomes then fuse with lysosomes, where enzymes degrade the mitochondria, thereby eliminating these defective organelles. The process of autophagy triggered by mitochondrial damage is regulated by a complex network involving multiple signaling pathways and proteins. For example, the PINK1/Parkin pathway is one of the most crucial signaling pathways in mitophagy. When mitochondrial damage occurs, PINK1 accumulates on the outer mitochondrial membrane and recruits the Parkin protein. Parkin then promotes the aggregation of autophagy-related proteins and the formation of autophagosomes, thereby initiating the mitophagy process.

At present, there is relatively little research on the role of mitochondrial autophagy in duodenal abnormalities. Some studies suggest that mitochondrial autophagy may be related to factors such as oxidative stress, inflammatory mediators, immune cells, and the intestinal mucosal barrier (15).

When mitochondrial dysfunction occurs, its outer membrane permeability increases, and apoptosis-related proteins distributed between the inner and outer membranes of mitochondria diffuse to the cytoplasm, thereby initiating the cell apoptosis program. This process may lead to an increase in the expression of inflammatory factors, thereby exacerbating intestinal inflammation, which may be the basis of gastrointestinal dysfunction (87).

Oxidative stress (OS) refers to the process in which the redox balance within a cell or organism is disrupted, resulting in the production of excessive oxygen or anaerobic free radicals, thereby causing damage to biomolecules within the cell. Mitochondria generate a large amount of ROS through their electron transport chain (ETC) and NADPH oxidase (NOX), which is the main triggering factor for OS (88). ROS include superoxide anions ( $O_{2}^{\cdot -}$ ), hydroxyl radicals ( ·OH ), and hydrogen peroxide (H_2_O_2_) (89). Under normal physiological conditions, ROS, as “oxidation–reduction messengers”, are at low levels within the body, participating in intracellular signal transduction and playing an important role in maintaining cell cycle, gene expression, and homeostasis of the intracellular environment (90).

**Figure 3:**
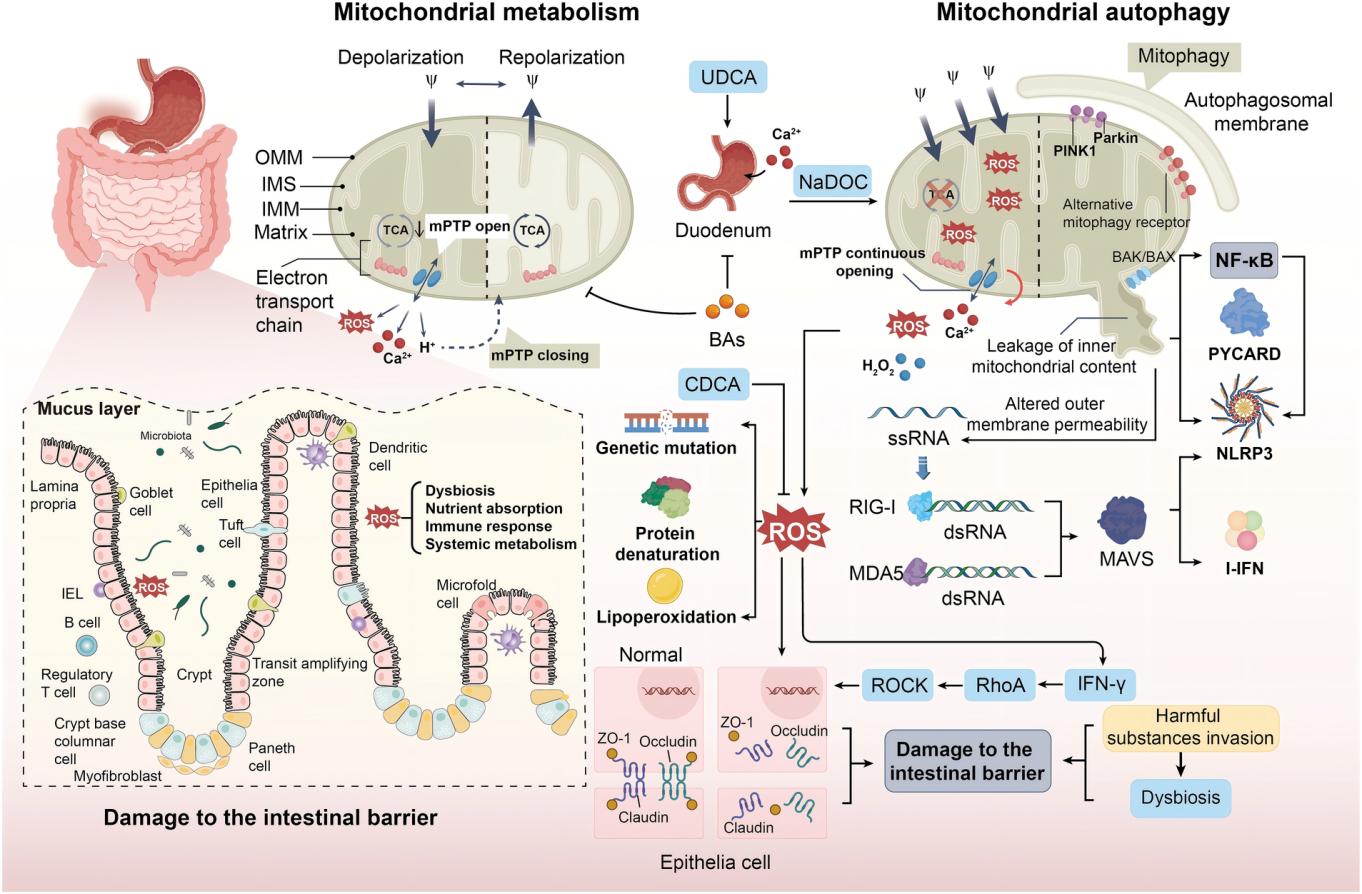
The role of mitochondrial autophagy in duodenal abnormalities. OS disrupts the cellular redox balance, leading to excessive ROS production and affecting biomolecules, including mitochondria generating ROS via ETC and NOX. Under normal conditions, ROS serves as signaling molecules to maintain homeostasis. However, conditions like hypoxia, nutrient deprivation, or IRI result in ROS accumulation, activating inflammation and causing gene mutations and protein misfolding, leading to structural and functional changes in intestinal epithelial cells. MPTP alters mitochondrial permeability and membrane potential during cell death; this process is influenced by factors such as Ca^2+^ overload and elevated ROS levels. ROS stimulate the production of inflammatory factors via the NF-κB pathway, leading to intestinal dysfunction. Additionally, BAs regulate intestinal Ca^2+^ absorption by modulating mitochondrial autophagy. Retinoic acid-inducible gene protein receptors and RLRs recognize mitochondrial-released RNA, inducing inflammatory responses. PRRs like TLR9 and RAGE identify mtDNA as pathogen-associated or damage-associated signals, triggering immune responses and inflammation that impair intestinal function and structure.

However, when a body experiences hypoxia, nutritional starvation, ischemia-reperfusion injury (IRI), or metabolic stress, mitochondrial fusion and fission are inhibited (91). This inhibition impedes normal metabolic functions, leading to increased production of ROS. The capacity of the antioxidant system may be insufficient to counteract this increase, resulting in oxidative stress. Excessive ROS can attack various intracellular biomolecules, damaging the structure and function of lipids, proteins, and DNA; triggering inflammation; and further exacerbating mitochondrial damage. This, in turn, affects normal cellular metabolism and function, potentially leading to apoptosis or necrosis of gastrointestinal epithelial cells. Moreover, ROS can induce gene mutations, protein denaturation, and lipid peroxidation, altering the structure and function of gastrointestinal epithelial cells (92) (Figure 3). Many components of the intestinal mucosal barrier, such as intestinal epithelial cells and the mucus layer, are particularly susceptible to ROS attack.

Mitochondrial permeability transition pore (MPTP) is a non-specific channel located between the inner and outer membranes of mitochondria. MPTP can alter mitochondrial permeability and membrane potential during cell death (93). This sustained pore activation is caused by mitochondrial Ca^2+^ overload, mitochondrial glutathione oxidation, elevated ROS levels, and other proapoptotic conditions (94). When an inflammatory response occurs, mitochondrial Ca^2+^ overload triggers the opening of MPTP, further exacerbating Ca^2+^ overload and promoting the production of ROS. ROS further enhances Ca^2+^ influx, and the two factors interact to maintain the open state of MPTP (95) (Figure 3). After mitochondrial damage, various components such as mitochondrial DNA (mtDNA) and other molecules are released, which are perceived as “danger signals” within the cytoplasm, subsequently triggering immune cell responses. mtDNA that is released into the cytoplasm can be recognized by intracellular receptors, activating a series of signaling pathways that lead to the activation of immune cells and the production of inflammatory mediators. When mitochondria are damaged, the main inflammasome NLRP3 and the apoptosis-associated spot-like protein (PYCARD) are relocated to the mitochondria-associated endoplasmic reticulum, causing an inflammatory response (96) (Figure 3). Cytokines and chemokines, as inflammatory mediators, attract and activate more immune cells to the site of damage. These immune cells, including macrophages, natural killer cells, and T cells, further exacerbate the inflammatory response in the duodenum and attempt to clear the damaged mitochondria and cells. However, excessive inflammation can also lead to tissue damage and the development of disease.

Intestinal tight junction proteins play an important role in maintaining the integrity and normal function of the intestinal mucosal barrier structure. The intestinal mucosal barrier is primarily composed of the intestinal epithelial cell layer, the mucus layer, and the gut microbiota. It plays a crucial role in preventing harmful substances, such as bacteria and toxins, from passing through the intestinal mucosa into other tissues, organs, and the bloodstream. When oxidative stress occurs, unsaturated fatty acids in cell membrane phospholipids undergo peroxidation after being attacked by ROS, changing the structure of zonula occlude-1 (ZO-1). Furthermore, claudin-4 and occludin are separated from the intestinal epithelial tight junction complex, leading to cellular dysfunction and disruption of the intestinal mucosal barrier function (97) (Figure 3).

Additionally, ROS can promote the secretion of IFN-γ, which activates Ras homolog gene family member A (RhoA), thereby upregulating Rho-associated coiled-coil containing protein kinase (ROCK) (98). This ultimately leads to the degradation of tight junction proteins, resulting in intestinal barrier dysfunction and compromising the normal function of the intestinal mucosal barrier. When the intestinal mucosal barrier is damaged, its ability to prevent harmful substances from passing through the intestinal mucosa into the body is diminished, potentially leading to intestinal inflammation, infections, and other related diseases. Moreover, the increased permeability due to barrier damage allows for the unchecked invasion of harmful substances, severely disrupting the balance of the gut microbiota and further exacerbating intestinal inflammation and infection. Concurrently, the oxidative products diffuse through the intestinal epithelium, the oxidation potential of the epithelium increases, and a large number of aerobic bacteria grow (99) (Figure 3). Studies also suggest that ROS can directly affect the biodiversity, classification, and function of bacteria, thereby altering the composition of gut microbiota and further impairing host nutrient absorption, immune response, and systemic metabolism (61). In addition, other mitochondrial components, such as the second mitochondrial-derived activator of caspase (Smac), mitochondrial N-formyl peptides (NFPs), cardiolipin (CL), and cytochrome c, have also been found to trigger inflammatory responses (100). In these processes, ROS, acting through the NF-κB pathway, stimulates the production of inflammatory factors, such as IL-6, leading to an increase in facultative anaerobic bacteria and intestinal dysfunction (101). Therefore, mitochondrial autophagy may play an important role in maintaining duodenal intestinal homeostasis, providing a new possible approach for treating FD, which is to regulate mitochondrial autophagy, eliminate excess ROS, prevent damage to the intestinal barrier function, maintain the stability of the intestinal microbiota, and thus maintain normal intestinal function.

In early studies, it was found that BAs that interfere with mitochondrial metabolism may inhibit the uptake of Ca^2+^ in the duodenum (102), preliminarily elucidating the relationship between duodenal abnormalities and mitochondrial autophagy. A study conducted in-depth experiments and found that sodium deoxycholate affects the uptake of Ca^2+^ in the duodenum by inducing oxidative or nitration stress, promoting apoptosis, and other pathways (103). Ursodeoxycholic acid (UDCA) enhances the uptake capacity of cations in the duodenum by increasing the activity of isocitrate dehydrogenase (IDH) in the tricarboxylic acid cycle and complex II in ETC. A study found that chenodeoxycholic acid (CDCA) upregulates the expression of genes related to cell cycle progression to increase ΔΨm and antioxidant capacity, reduce intracellular ROS and MDA levels, regulate mitochondrial autophagy, and promote intestinal epithelial cell proliferation (104) (Figure 3). These studies confirm that BAs affect the normal physiological function of the duodenum, especially the uptake of Ca^2+^, by regulating mitochondrial autophagy.

Mitochondrial dysfunction and changes in outer membrane permeability can also lead to RNA release from mitochondria (105). Retinoic-acid-inducible gene I (RIG-I) and melanoma differentiation-associated antigen 5 (MDA5) are important members of the RIG-I-like receptors (RLRs) family of retinoic acid-inducible genes. After recognizing and binding to double stranded 5′-triphosphate RNA (3pRNA) (106), the two proteins stimulate the adapter protein of mitochondrial antiviral signaling proteins (MAVS) to induce pro-inflammatory cytokines and regulate the production of type I interferon (I-IFN), promoting an inflammatory response (107) (Figure 3).

A recent study demonstrated that in addition to the aforementioned pathways, Toll-like receptor 9 (TLR9) and pattern recognition receptors (PRRs), such as RAGE, recognize mitochondrial DNA (mtDNA) as pathogens or injury signals, triggering immune and inflammatory responses that may damage the function and structure of the duodenum (108) (Figure 3). Although some studies have been reported, this mechanism has not been fully elucidated. Further experiments are needed to gain a deeper understanding.

## 6 Summary and outlook

In summary, from the perspective of mitochondrial autophagy, we explored in detail the multi-level mechanisms of mucosal barrier damage, release of inflammatory factors, and disruption of intracellular signal transduction, thereby gaining an in-depth understanding of the occurrence and developmental process of duodenal abnormalities. Mitochondrial autophagy is not only a cellular protective mechanism but also a regulator of intracellular balance and metabolic activity. Mitochondrial autophagy plays a crucial role in regulating intracellular energy balance by clearing excess or dysfunctional mitochondria, providing a new research perspective for duodenal abnormalities.

Mitochondrial autophagy is crucial in regulating oxidative stress, the expression of inflammatory factors, and intestinal barrier function. The increase in oxidative stress is often accompanied by mitochondrial dysfunction, leading to excessive ROS generation and subsequently triggering an inflammatory response. This process may have a negative impact on the normal function of the duodenum, thereby exacerbating the pathological process of FD. The regulation of mitochondrial autophagy is closely related to the integrity of the intestinal mucosal barrier. Dyspepsia is a symptom caused by abnormal gastric motility, which is caused by harmful stimuli transmitted from the duodenal mucosa through the nerves (109). Compared with healthy individuals, patients with FD have duodenums that are more sensitive to stimulation and are more prone to digestive symptoms. Research has confirmed that microinflammation and increased permeability in duodenal mucosa are fundamental causes of FD (110).

Despite some progress, issues remain that require urgent attention. The interrelationships between mitochondrial autophagy and various reactions, such as inflammation and immune response, have not yet been fully elucidated. Further studies are needed in the future to clarify the molecular mechanisms and signaling pathways of mitochondrial autophagy regulation in duodenal abnormalities and related pathological reactions. Drug intervention studies targeting mitochondrial autophagy should aim to modify the abnormal mitochondrial function in the duodenum of patients with FD, study the specific mechanism of drug action, and provide a theoretical basis for related treatment strategies, thereby improving symptoms and preventing disease progression.
